# Supplementary material for: HIVprotI: an integrated web based platform for prediction and design of HIV proteins inhibitors
Source: J Cheminform. 2018 Mar 9;10:12. doi: 10.1186/s13321-018-0266-y (PMC5845081; doi:10.1186/s13321-018-0266-y)
Supplement: Supplementary file 2 — Additional file 2. Source code of HIVProtI web server. [file 13321_2018_266_MOESM2_ESM.zip › HIVprotI_Source-code/hivprotI/weka-3-6-6/doc/weka/gui/SimpleDateFormatEditor.html]

SimpleDateFormatEditor


---


|  |  |  |
| --- | --- | --- |
| **Overview** \| **Package** \| **Class** \| **Tree** \| **Deprecated** \| **Index** \| **Help** \| **Weka's home** | |  |
| **PREV CLASS**   **NEXT CLASS** | **FRAMES**    **NO FRAMES**     **All Classes** |  |
| SUMMARY: NESTED \| FIELD \| CONSTR \| METHOD | DETAIL: FIELD \| CONSTR \| METHOD |  |


---


## weka.gui Class SimpleDateFormatEditor

```
java.lang.Object
  weka.gui.SimpleDateFormatEditor
```

**All Implemented Interfaces:**: java.beans.PropertyEditor

---

``` public class SimpleDateFormatEditor extends java.lang.Object implements java.beans.PropertyEditor ```

Class for editing SimpleDateFormat strings.

**Version:**
:   $Revision: 7059 $

**Author:**
:   FracPete (fracpete at waikato dot ac dot nz)

**See Also:**: `SimpleDateFormat`

---

| **Field Summary** | |
| --- | --- |
| `static java.lang.String` | `DEFAULT_FORMAT`             the default format |


**Constructor Summary**

`SimpleDateFormatEditor()`
  
          Constructs a new SimpleDateFormatEditor.


| **Method Summary** | |
| --- | --- |
| `void` | `addPropertyChangeListener(java.beans.PropertyChangeListener listener)`             Adds an object to the list of those that wish to be informed when the date format changes. |
| `java.lang.String` | `getAsText()`             Returns the date format string. |
| `java.awt.Component` | `getCustomEditor()`             Gets a GUI component with which the user can edit the date format. |
| `java.lang.String` | `getJavaInitializationString()`             Returns the Java code that generates an object the same as the one being edited. |
| `java.lang.String[]` | `getTags()`             Some objects can return tags, but a date format cannot. |
| `java.lang.Object` | `getValue()`             Gets the date format that is being edited. |
| `boolean` | `isPaintable()`             Indicates whether the object can be represented graphically. |
| `void` | `paintValue(java.awt.Graphics gfx, java.awt.Rectangle box)`             Paints a graphical representation of the object. |
| `void` | `removePropertyChangeListener(java.beans.PropertyChangeListener listener)`             Removes an object from the list of those that wish to be informed when the date format changes. |
| `void` | `setAsText(java.lang.String text)`             Sets the date format string. |
| `void` | `setValue(java.lang.Object value)`             Sets the value of the date format to be edited. |
| `boolean` | `supportsCustomEditor()`             Indicates whether the date format can be edited in a GUI, which it can. |

**Methods inherited from class java.lang.Object**

`equals, getClass, hashCode, notify, notifyAll, toString, wait, wait, wait`

**Field Detail**

### DEFAULT\_FORMAT

```
public static final java.lang.String DEFAULT_FORMAT
```

:   the default format

    **See Also:**: Constant Field Values


**Constructor Detail**

### SimpleDateFormatEditor

```
public SimpleDateFormatEditor()
```

:   Constructs a new SimpleDateFormatEditor.


**Method Detail**

### setValue

```
public void setValue(java.lang.Object value)
```

:   Sets the value of the date format to be edited.

    :   **Specified by:**: `setValue` in interface `java.beans.PropertyEditor`
    :   **Parameters:**: `value` - a SimpleDateFormat object to be edited

---


### getValue

```
public java.lang.Object getValue()
```

:   Gets the date format that is being edited.

    :   **Specified by:**: `getValue` in interface `java.beans.PropertyEditor`
    :   **Returns:**: the edited SimpleDateFormat object

---


### isPaintable

```
public boolean isPaintable()
```

:   Indicates whether the object can be represented graphically. In this case
    it can.

    :   **Specified by:**: `isPaintable` in interface `java.beans.PropertyEditor`
    :   **Returns:**: true

---


### paintValue

```
public void paintValue(java.awt.Graphics gfx,
                       java.awt.Rectangle box)
```

:   Paints a graphical representation of the object. It just prints the
    format.

    :   **Specified by:**: `paintValue` in interface `java.beans.PropertyEditor`
    :   **Parameters:**: `gfx` - the graphics context to draw the representation to: `box` - the bounds within which the representation should fit.

---


### getJavaInitializationString

```
public java.lang.String getJavaInitializationString()
```

:   Returns the Java code that generates an object the same as the one being edited.

    :   **Specified by:**: `getJavaInitializationString` in interface `java.beans.PropertyEditor`
    :   **Returns:**: the initialization string

---


### getAsText

```
public java.lang.String getAsText()
```

:   Returns the date format string.

    :   **Specified by:**: `getAsText` in interface `java.beans.PropertyEditor`
    :   **Returns:**: the date format string

---


### setAsText

```
public void setAsText(java.lang.String text)
```

:   Sets the date format string.

    :   **Specified by:**: `setAsText` in interface `java.beans.PropertyEditor`
    :   **Parameters:**: `text` - the date format string

---


### getTags

```
public java.lang.String[] getTags()
```

:   Some objects can return tags, but a date format cannot.

    :   **Specified by:**: `getTags` in interface `java.beans.PropertyEditor`
    :   **Returns:**: null

---


### getCustomEditor

```
public java.awt.Component getCustomEditor()
```

:   Gets a GUI component with which the user can edit the date format.

    :   **Specified by:**: `getCustomEditor` in interface `java.beans.PropertyEditor`
    :   **Returns:**: an editor GUI component

---


### supportsCustomEditor

```
public boolean supportsCustomEditor()
```

:   Indicates whether the date format can be edited in a GUI, which it can.

    :   **Specified by:**: `supportsCustomEditor` in interface `java.beans.PropertyEditor`
    :   **Returns:**: true

---


### addPropertyChangeListener

```
public void addPropertyChangeListener(java.beans.PropertyChangeListener listener)
```

:   Adds an object to the list of those that wish to be informed when the
    date format changes.

    :   **Specified by:**: `addPropertyChangeListener` in interface `java.beans.PropertyEditor`
    :   **Parameters:**: `listener` - a new listener to add to the list

---


### removePropertyChangeListener

```
public void removePropertyChangeListener(java.beans.PropertyChangeListener listener)
```

:   Removes an object from the list of those that wish to be informed when the
    date format changes.

    :   **Specified by:**: `removePropertyChangeListener` in interface `java.beans.PropertyEditor`
    :   **Parameters:**: `listener` - the listener to remove from the list


---


|  |  |  |
| --- | --- | --- |
| **Overview** \| **Package** \| **Class** \| **Tree** \| **Deprecated** \| **Index** \| **Help** \| **Weka's home** | |  |
| **PREV CLASS**   **NEXT CLASS** | **FRAMES**    **NO FRAMES**     **All Classes** |  |
| SUMMARY: NESTED \| FIELD \| CONSTR \| METHOD | DETAIL: FIELD \| CONSTR \| METHOD |  |


---
